# Leveraging off higher plant phylogenetic insights for antiplasmodial drug discovery

**DOI:** 10.1007/s13659-023-00396-x

**Published:** 2023-10-05

**Authors:** Phanankosi Moyo, Luke Invernizzi, Sephora M. Mianda, Wiehan Rudolph, Warren A. Andayi, Mingxun Wang, Neil R. Crouch, Vinesh J. Maharaj

**Affiliations:** 1 Biodiscovery Center, Department of Chemistry, Faculty of Natural and Agricultural Sciences, University of Pretoria, Private Bag X 20, Hatfield, Pretoria, 0028 South Africa; 2 Department of Physical and Biological Sciences, Murang’a University of Technology, Murang’a, Kenya; 3 Computer Science and Engineering, University of California Riverside, 900 University Ave, Riverside, CA 92521 USA; 4 Biodiversity Research and Monitoring Directorate, South African National Biodiversity Institute, Berea Road, P.O. Box 52099, Durban, 4007 South Africa; 5 School of Chemistry and Physics, University of KwaZulu-Natal, Durban, 4041 South Africa

**Keywords:** Natural products, Plants, Phylogenetics, Malaria, Drug-resistance, ‘Hot’ plants

## Abstract

The antimalarial drug-resistance conundrum which threatens to reverse the great strides taken to curb the malaria scourge warrants an urgent need to find novel chemical scaffolds to serve as templates for the development of new antimalarial drugs. Plants represent a viable alternative source for the discovery of unique potential antiplasmodial chemical scaffolds. To expedite the discovery of new antiplasmodial compounds from plants, the aim of this study was to use phylogenetic analysis to identify higher plant orders and families that can be rationally prioritised for antimalarial drug discovery. We queried the PubMed database for publications documenting antiplasmodial properties of natural compounds isolated from higher plants. Thereafter, we manually collated compounds reported along with plant species of origin and relevant pharmacological data. We systematically assigned antiplasmodial-associated plant species into recognised families and orders, and then computed the resistance index, selectivity index and physicochemical properties of the compounds from each taxonomic group. Correlating the generated phylogenetic trees and the biological data of each clade allowed for the identification of 3 ‘hot’ plant orders and families. The top 3 ranked plant orders were the (i) Caryophyllales, (ii) Buxales, and (iii) Chloranthales. The top 3 ranked plant families were the (i) Ancistrocladaceae, (ii) Simaroubaceae, and (iii) Buxaceae. The highly active natural compounds (IC_50_ ≤ 1 µM) isolated from these plant orders and families are structurally unique to the ‘legacy’ antimalarial drugs. Our study was able to identify the most prolific taxa at order and family rank that we propose be prioritised in the search for potent, safe and drug-like antimalarial molecules.

## Introduction

Malaria is a vector-borne tropical disease caused by unicellular protozoan parasites of the genus *Plasmodium* [1]. Despite conspicuous progress in controlling and managing malaria, the disease remains a serious public health challenge. Malaria is currently endemic in 84 countries, with the World Health Organisation (WHO) reporting the African Region as the most afflicted by this disease [2]. In 2021 there was a total of 247 million clinical malaria cases and 619,000 malaria-induced fatalities globally, with the African Region accounting for 95% of the reported cases and fatalities. Current antimalarial drugs are becoming less effective due to the emergence and spread of drug-refractory *Plasmodium* parasite strains [3]. These strains arise as a product of mutations, most notably due to either DNA replication errors or damage induced by reactive oxygen species [4, 5]. These mutations give rise to phenotypes with changes in, for example, either drug targets or transporters which confer *Plasmodium* parasites with resistance to antimalarial drugs. This resistance phenomenon has been observed for all previously used antimalarials [6], including the current WHO-recommended first-line treatment drugs for malaria, namely artemisinin-based combination therapy (ACT) [6]. This highlights the need to discover and develop new and alternative malaria treatment regimens.

One promising strategy for discovering new curative antimalarial compounds is the identification of plants that produce compounds with antiplasmodial activity. Plants have evolved to produce a diverse array of chemical compounds to defend themselves against pathogens and parasites, many of which have potential medicinal properties. This has made them a reliable source for the discovery of privileged chemical scaffolds, which have served as a foundation for developing a plethora of pharmaceutical agents [7]. Some of the linchpin malaria chemotherapeutics were likewise discovered from plants. From a *Cinchona* species (Rubiaceae), the alkaloid quinine was isolated. This compound served as a template from which derivatives, including chloroquine, were synthesised. Lapachol, a naphthoquinone first isolated in 1882 from the bark of *Tabebuia avellanedae* (Bignoniaceae), served as a scaffold which inspired the development of the antimalarial drug atovaquone. Similarly, from the Chinese herb *Artemisia annua* (Asteraceae), the sesquiterpene lactone artemisinin was isolated and semi-synthesised to form prolific fast-acting derivatives, namely artemether, dihydroartemisinin and artesunate which are the core constituents of the ACT regimen [8]. Over the last century, these plant-derived antimalarials have saved millions of lives [9–11]. In view of this, there is merit in the continued investigation of plants in search of novel antimalarial agents to redress the drug-resistance scourge.

Given the diversity and expansiveness of the plant kingdom (*ca*. 370,000 flowering plant species [12]) and limited research resources, there is a need for a rational strategy to streamline and focus drug screening projects on selected plant species. Adoption of such strategies is envisaged to expedite antimalarial drug discovery by simplifying the plant screening process. Moreover, this is anticipated to come with the added advantage of an increased likelihood of discovering promising leads (hits) as research resources will be focused on the ‘hot’ taxonomic groups (i.e., taxa with an overrepresentation of active compounds) [13, 14]. One logical approach adopted in some drug discovery projects that prioritise plant subjects for evaluation, is phylogenetic analysis [15–17]. Such phylogenetic analyses allow for identification of ‘hot’ plant genera, families or orders that have been demonstrated to produce bioactive compounds against specific therapeutic targets [18]. This concept emanates from the premise that phylogeny and biosynthetic pathways are correlated; therefore, the production of specific bioactive natural products with peculiar biological properties will likely be common to closely related plant species at the level of genus, family, or families within an order [14]. In line with this principle various members of the filamentous bacterial genus *Streptomyces* have yielded an array of secondary metabolites (including tetracyclines, aminoglycosides and macrolides) with commercially useful antimicrobial activity [19, 20]. Similarly, amongst higher plants, the Amaryllidaceae plant family is well established to exclusively produce specific alkaloids, including the lycorine-type alkaloids, which exhibit a distinct pharmacological profile [21]. The correlation of phylogenetic analysis and pharmacological data allows us to effectively identify ‘hot’ plant taxonomic groups of specific interest to man based on what is experimentally known about compounds isolated from plant species in those plant orders and families. From these taxonomic groups, closely related, untapped species can be rationally prioritised for pharmacological evaluation [18].

Over recent decades, numerous natural compounds isolated from different plant species have been evaluated for their in vitro antiplasmodial activity. This study aimed to use phylogenetic analysis to identify ‘hot’ plant orders, and families that: (i) produce active antiplasmodial compounds (IC_50_ ≤ 10 µM) with (ii) acceptable resistance index (RI ≤ 10), (iii) a selectivity index (SI ≥ 10), and (iv) drug-like properties. We collated data on active and inactive antiplasmodial plant-derived compounds from literature published between 1964 and 2021. We determined, including through resolution of synonymy, the identity of plant species yielding antiplasmodial isolates and used the information to construct phylogenetic trees, which were correlated to quantified antiplasmodial and cytotoxicity data. From the generated trees, we were able to establish the distribution patterns of plant species in different ‘hot’ plant families and orders. We believe this analysis will facilitate the selection of taxa warranting further evaluation in antimalarial drug discovery programs, to optimize project outcomes.

## Results, discussion and conclusion

### Descriptive analysis: articles evaluated, plant taxonomy, and antiplasmodial screening

**Fig. 1:**
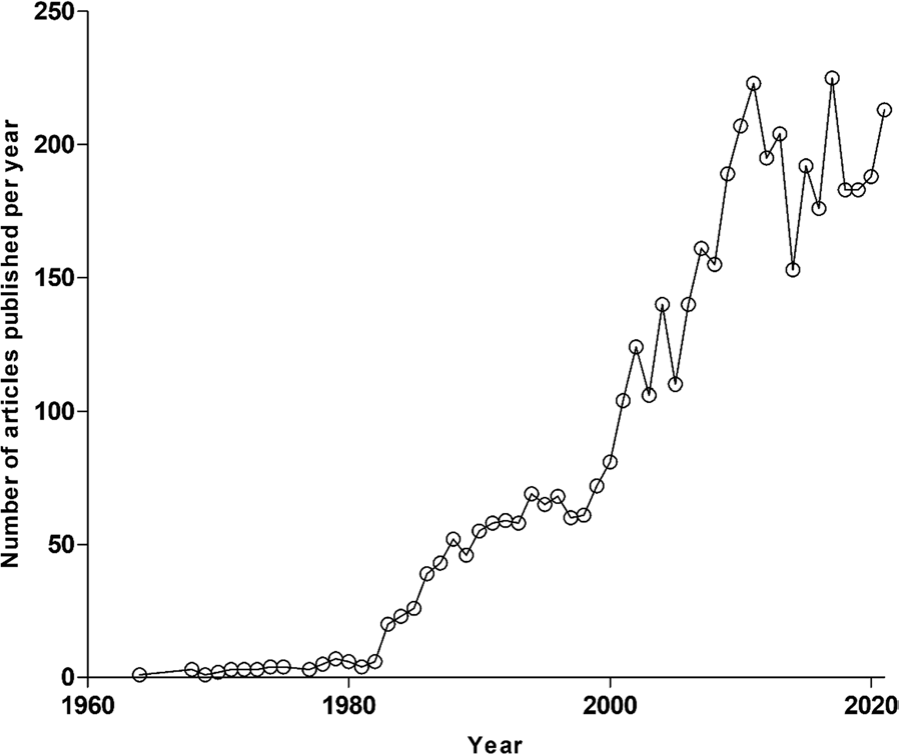
Number of articles published per year (1964 to 2021) on “*Plasmodium falciparum* and natural product”. The annual number of publications on the topic of “*Plasmodium falciparum* and natural product” has evidently increased over the years within the last decade, being the most productive in that regard. These search results were realised following the query made on PubMed and limited to abstracts

A PubMed database query using the key phrase “*Plasmodium falciparum* and natural product”, limiting the search to the abstract, yielded a total of 3863 articles (Fig. 1). These articles were published within a 58-year period ranging from 1964 to 2021 (Fig. 1). From this pool of articles, duplicates and publications reporting on non-higher plant-derived natural products were excluded. Furthermore, studies where natural compounds were neither isolated nor screened in vitro against *P. falciparum*, were excluded. Following the application of these exclusion criteria, we filtered to a total subset of 455 articles.

From the relevant 455 articles, 2426 natural compounds, reportedly either active or inactive in vitro against *P. falciparum* parasites were manually compiled. These compounds were collected along with components of plant species from which they were initially isolated, and their respective antiplasmodial activities were determined. These molecules were isolated from 439 plant species belonging to 99 vascular plant families referred to 37 plant orders (Table 1). This total number of plant species only represents a small fraction (*ca.* 0.1%) of all known higher plant species worldwide. The *ca.* 2400 plant-derived compounds which our study was limited to is substantially less than that analysed in a similar study by Zhu et al. [15] where *ca.* 31,000 compounds were considered. In their study, Zhu and colleagues examined the species-distribution of 939 clinically approved natural product-derived drugs, 369 clinical candidates, and 119 preclinical candidates. Furthermore, 13,548 marine derived natural products and 19,721 bioactive secondary metabolites were included in their study [15]. The natural product derived drugs and bioactive compounds considered were from several sources including plants, microorganisms, and marine organisms [15]. Furthermore, these were drugs and bioactive compounds for several therapeutic areas [15]. Disease-area focused investigations have previously analysed the number of compounds consistent with our study, although not for antiplasmodial activity. For example, in their investigation to examine the phylogenetic distribution of anticancer drugs, Li et al*.* [22] analysed 207 natural product derived drugs. From a malaria drug discovery standpoint our study is, to the best of our knowledge, the first to extensively examine the relationship between phylogeny and antiplasmodial activity of natural product compounds. Earlier approaches have focused on compound classes in relation to antiplasmodial activity, exampled by the study of Egieyeh et al. [23] which used cheminformatics profiling to prioritise natural products for antimalarial drug discovery. In their study they managed to analyse 1040 natural product compounds isolated from plants, microorganisms, and marine sources [23].

**Table 1:** Taxonomic representation of plant species yielding compounds subsequently investigated for antiplasmodial activity, arranged by Order

Plant Order	No. of accepted^a^		No. of investigated (*ca.*)^b^		% Investigated^c^	
	Species	Families	Species	Families	Species	Families
Alismatales	4476	14	1	1	0.02	7.14
Apiales	5935	7	5	1	0.08	14.29
Asparagales	39,041	14	9	3	0.02	21.43
Asterales	37,448	11	48	1	0.13	9.09
Boraginales	3522	1	1	1	0.03	100.00
Brassicales	5172	17	2	3	0.04	17.65
Buxales	130	1	2	1	1.54	100.00
Canellales	114	2	2	2	1.75	100.00
Caryophyllales	12,797	41	13	6	0.10	14.63
Celastrales	1385	2	6	1	0.43	50.00
Chloranthales	73	1	3	1	4.11	100.00
Commelinales	928	5	1	1	0.11	20.00
Cornales	707	7	2	2	0.28	28.57
Cucurbitales	3368	8	1	1	0.03	12.50
Cupressales	222	3	4	2	1.80	66.67
Dioscoreales	896	3	1	1	0.11	33.33
Dipsacales	1348	2	1	1	0.07	50.00
Ericales	15,376	22	6	4	0.04	18.18
Fabales	25,024	4	35	1	0.14	25.00
Fagales	1629	7	6	3	0.37	42.86
Gentianales	23,061	5	55	5	0.24	100.00
Lamiales	28,037	24	27	9	0.10	37.50
Laurales	3831	7	8	3	0.21	42.86
Lycopodiales	425	1	1	1	0.24	100.00
Magnoliales	3228	6	21	3	0.65	50.00
Malpighiales	19,119	36	45	11	0.24	30.56
Malvales	7404	10	5	3	0.07	30.00
Myrtales	14,510	9	17	3	0.12	33.33
Oxalidales	2093	7	3	3	0.14	42.86
Piperales	4276	3	8	2	0.19	66.67
Poales	25,176	14	6	2	0.02	14.29
Proteales	2027	4	2	2	0.10	50.00
Ranunculales	6091	7	13	4	0.21	57.14
Rosales	10,894	9	7	3	0.06	33.33
Santalales	2513	7	2	1	0.08	14.29
Sapindales	6826	9	65	6	0.95	66.67
Zingiberales	2971	8	5	1	0.17	12.50

^a^The No. of accepted plant species and families per plant order are as reported in the World Flora Online Plant List database (https://wfoplantlist.org/plant-list/), which was also sourced to resolve issues of synonymy and nomenclature at all taxonomic ranks

^b^The total no. of investigated plant species and families per plant order are from data collated in this study

^c^The % investigated for each order was calculated by dividing the No. of investigated species and families by the No. of accepted species and families, respectively

Of the 2400 compounds analysed in our study, the Asterids and Rosid lineages were the most overrepresented clades with 6 and 10 plant orders, respectively (see Table 3). The plant order with the greatest number of different accepted plant species investigated was the Sapindales (n = 65), closely followed by Asterales (n = 48) and Gentianales (n = 55). The Chloranthales (4%), Cupressales (1.8%), Canellales (1.75%), Buxales (1.54%), and Sapindales (0.95%) were the most investigated relative to the total number of accepted plant species known to occur in those orders. In contrast, the Asparagales (0.02%), and Alismatales (0.02%) were evidently the orders least investigated, considering their species richness (Table 1).

Compounds isolated from plant species in the different taxonomic groups were primarily assessed for activity against the 3D7 (n = 426), D6 (n = 254), and NF54 (n = 182) intra-erythrocytic asexual *P. falciparum* parasite drug-sensitive (D-S) strains. In vitro evaluation of potency against the intra-erythrocytic asexual *P. falciparum* parasite drug-resistant (D-R) parasites was predominantly carried out on the K1 (n = 689), W2 (n = 399) and Dd2 (n = 317) strains (Table 2).

**Table 2:** Top 5 D-S and D-R intra-erythrocytic asexual *P. falciparum* parasite strains most targeted for in vitro antiplasmodial screening of plant-derived compounds in reports considered

*P. falciparum* strain (D-S)	No. of compounds screened	*P. falciparum* strain (D-R)	No. of compounds screened
3D7	403	K1	672
D6	257	W2	372
NF54	183	Dd2	295
D10	98	FCB1	189
FCA	81	FCM29	54

### Phylogenetic analyses correlated to biological data

**Fig. 2:**
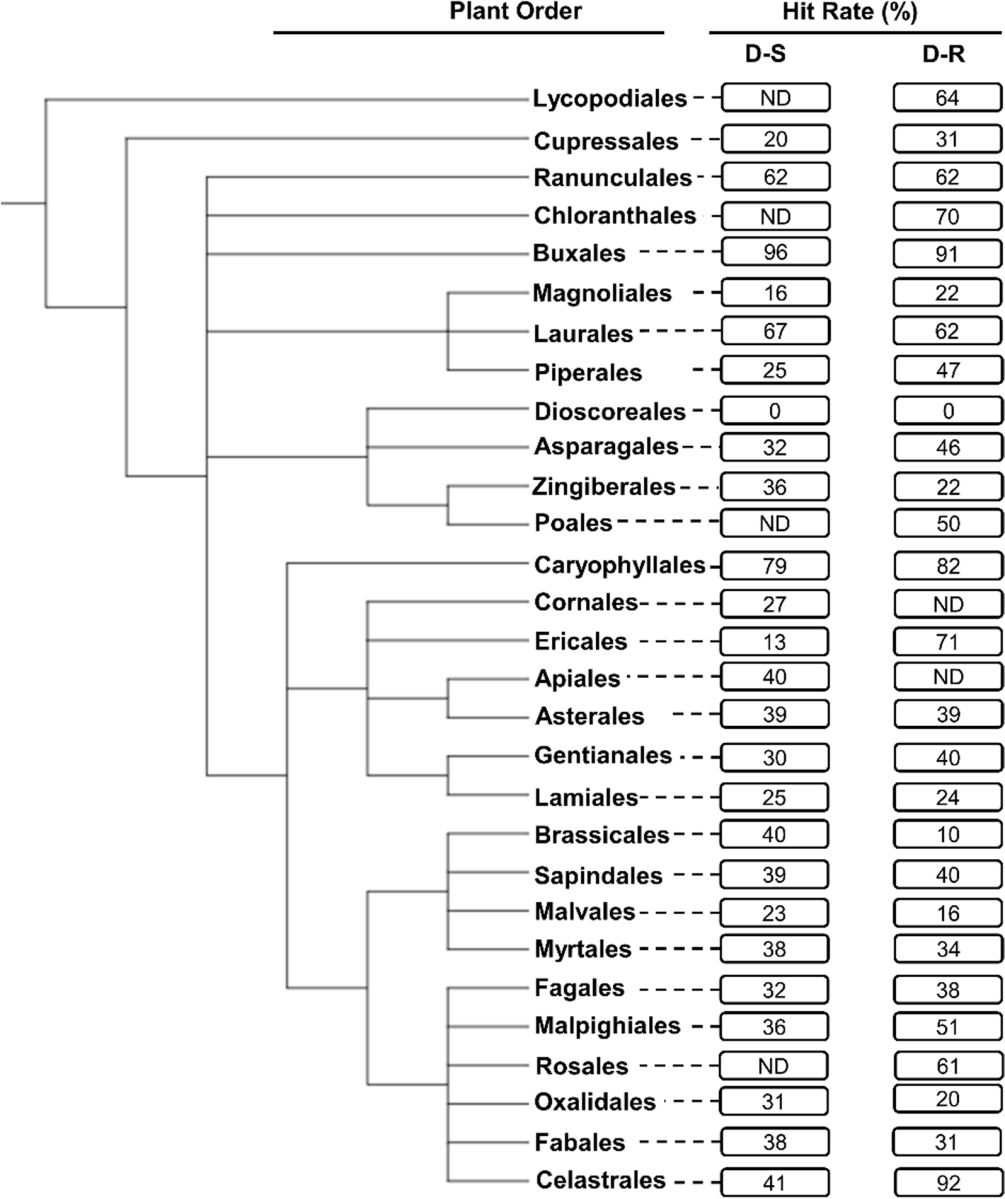
Phylogenetic tree of plant orders investigated in vitro for their activity against intra-erythrocytic asexual *P. falciparum* parasites. The tree was generated using NCBI Taxonomy and processed using iTOL. *ND* not determined: This applies for plant orders with < 10 compounds isolated from them and subsequently evaluated for their antiplasmodial activity. The hit rate (HR) is the % of compounds with an IC_50_ ≤ 10 µM for each plant order. D-S—drug-sensitive. D-R—drug-resistant

**Fig. 3:**
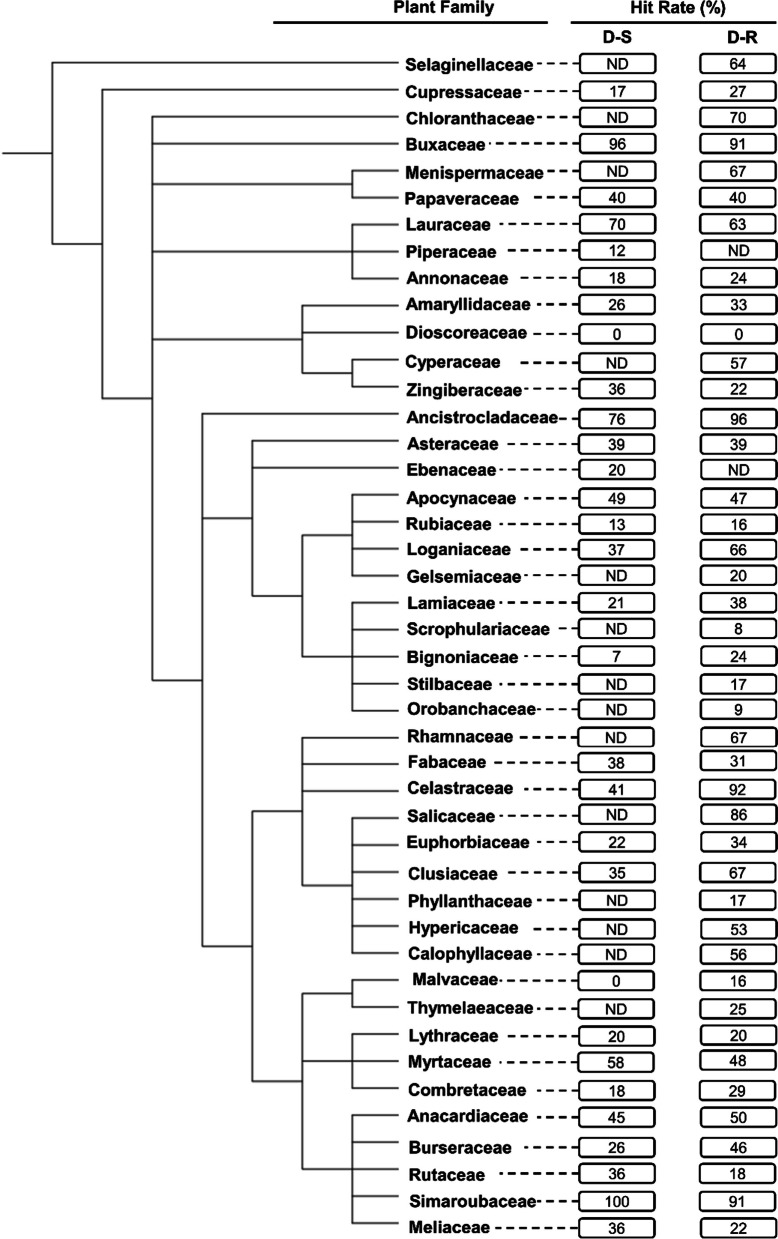
Phylogenetic tree of plant families investigated in vitro for their activity against the intra-erythrocytic asexual *P. falciparum* parasites. The tree was generated using NCBI Taxonomy and processed using iTOL. *ND* not determined: This applies to plant families with < 10 compounds isolated from them and subsequently evaluated for their antiplasmodial activity. The hit rate (HR) is the % of compounds with an IC_50_ ≤ 10 µM for each plant family

Following identification to species rank of taxa yielding compounds tested for antiplasmodial activity phylogenetic trees (cladograms) of higher plant orders and plant families were constructed using the NCBI Taxonomy database [24] and graphically displayed using an online tool, viz., the ‘interactive Tree of Life’ (iTOL) (Figs. 2 and 3) [25]. The constructed trees are consistent with the taxonomic classification and nomenclature reflected in ‘The World Flora Online’ [26]. The relationship between antiplasmodial activity and phylogenetic relationships at order and associated family levels was then determined and expressed as ‘’hit rates (HR) %’’ for taxonomic groups with ≥ 10 compounds isolated from them. The HR were calculated by dividing the number of compounds with an IC_50_ ≤ 10 µM by the total number of compounds isolated and experimentally evaluated for activity against either the D-S or D-R plasmodia. Plant taxa were correlated to the calculated HR of their compounds. Given the extensive diversity of higher plants globally, we consider it prudent to strategically focus discovery phase research on either ‘hot’ plant orders, or ‘hot’ families which yield compounds with high HR. This is assumed to increase the likelihood of successfully identifying active antiplasmodial compounds within a short time frame.

Generally, compounds from most plant orders showed high HR of 35% and 43% against asexual D-S and D-R *P. falciparum* parasites, respectively (Fig. 2). Compounds isolated from the Buxales had the highest HR (96%, n (number of compounds) = 25). This was closely followed by the Caryophyllales (HR = 79%, n = 53) and Laurales (HR = 67%, n = 12) (Fig. 2). The lowest HR were noted for the Dioscoreales (HR = 0%, n = 14), Ericales (HR = 13%, n = 23), and Magnoliales (HR = 16%, n = 91). Against D-R strains, the Celastrales (HR = 92%, n = 12) and Buxales (HR = 91%, n = 11) were found to have the highest HR, whereas the Dioscoreales (HR = 0%, n = 14), and Brassicales (HR = 10%, n = 10) presented the lowest HR against this *Plasmodium* form. Noteworthy plant orders with markedly different HR in relation to D-S and D-R parasites were the Celastrales (51% difference), Brassicales (30% difference) and Piperales (22% difference).

Compounds isolated from the Simaroubaceae (in Sapindales) demonstrated the highest HR (100%, n = 19) against the D-S parasites (Fig. 3). This plant family was closely followed by the Buxaceae (in Buxales) (HR = 96%, n = 25), Ancistrocladaceae (in Caryophyllales) (HR = 76%, n = 42), and Lauraceae (in Laurales) (HR = 70%, n = 10) (Fig. 3). The lowest HR were noted for the Dioscoreaceae (in Dioscoreales) (HR = 0%, n = 14), Malvaceae (in Malvales) (HR = 0%, n = 10), and Bignoniaceae (in Lamiales) (HR = 7%, n = 14). Against D-R strains, the Ancistrocladaceae (HR = 96%, n = 45) had the highest HR, marginally more than that for the Celastraceae (in Celastrales) (HR = 92%, n = 12), Buxaceae (HR = 91%, n = 11) and Simaroubaceae (HR = 91%, n = 55). The Dioscoreaceae (HR = 0%, n = 14), Scrophulariaceae (in Lamiales) (HR = 8%, n = 25), Rubiaceae (in Gentianales) (HR = 15%, n = 53), Phyllanthaceae (in Malpighiales) (HR = 16%, n = 18), and Malvaceae (HR = 16%, n = 19) demonstrated the lowest HR against the D-R parasites (Fig. 3). Plant families with notably different HR against the D-S and D-R parasites were Celastraceae (51% difference), and Loganiaceae (in Gentianales) (29% difference).

**Fig. 4:**
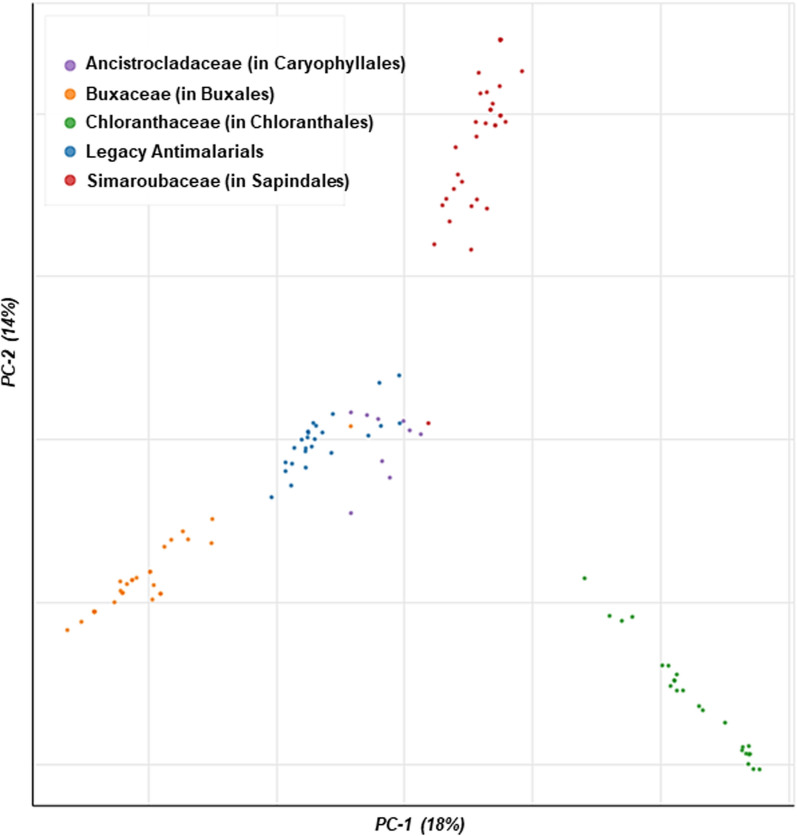
Launched drug chemical space of the ‘legacy’ antimalarials and natural product compounds isolated from the ‘hot’ plant orders and families. The online Python library for chemical space visualization ChemPlot [33], was used to launch the chemical space of the natural compounds and ‘legacy’ antimalarials

Overall, from this preliminary analysis, the Buxales and Caryophyllales have consistently emerged as the ‘hottest’ plant orders. The Simaroubaceae, Buxaceae, and Ancistrocladaceae have emerged as the ‘hottest’ plant families, routinely displaying high HR against both the D-S and D-R *Plasmodium* parasites (Fig. 4).

### Antiplasmodial activity and cytotoxicity of compounds isolated from different plant orders

To further assess the productivity of the different plant orders and families, we expanded our analysis by determining the % of compounds, in each plant order and family, classified as either highly active (HA) (IC_50_ ≤ 1 µM), moderately active (MA) (10 µM ≥ IC_50_ > 1 µM) or poorly active (PA) (IC_50_ > 10 µM) (Table 3). We anticipate that research that prioritises plant orders or families that produce mainly HA compounds, will yield more rewarding leads. Furthermore, considering the need to address the resistance phenomenon, we examined the resistance index (RI) of compounds as an indicator of their efficacy against the D-R strains relative to D-S *Plasmodium* parasite strains. Additionally, as a proxy indicator for the preference of compounds to compromise *Plasmodium* parasite proliferation ahead of that of mammalian cell lines, we assessed the selectivity index (SI) of the compounds investigated per plant order and family. We consider that plant orders and families producing compounds with an RI ≤ 10 and an SI ≥ 10 should be preferentially prioritised for investigation (as per guidelines provided by the Medicines for Malaria Venture (MMV)—https://www.mmv.org/).

**Table 3:**
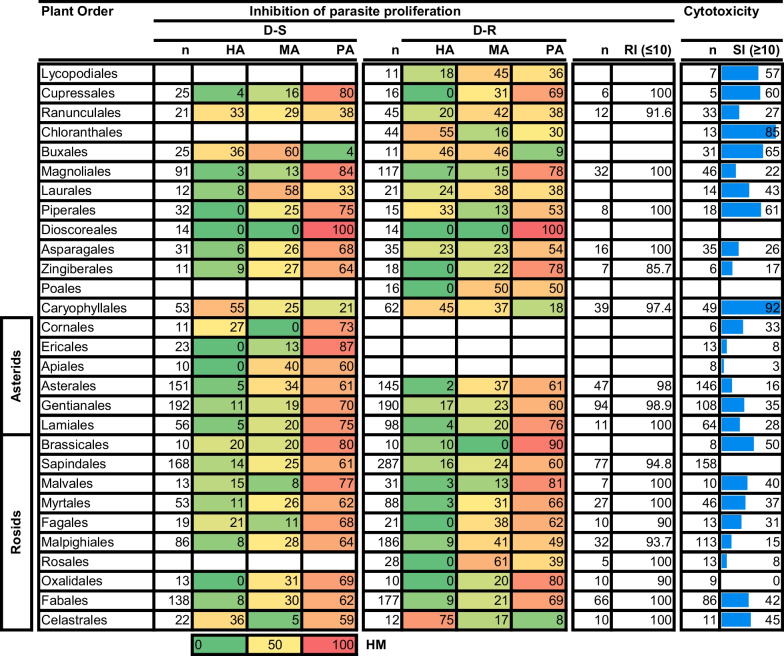
Antiplasmodial activity and cytotoxicity of compounds isolated from plants of different orders

n = number of compounds. HA, MA and PA values are expressed in % of total compounds evaluated. RI shows % of compounds with RI ≤ 10. SI shows % of compounds with SI ≥ 10. For RI and SI we have only presented data where ≥ 5 compounds have been evaluated and reported. The heat map (HM) ranges from green (lowest value, 0%) to yellow (mid-range value, 50%) to red (highest value, 100%), visually illustrating the proportion of compounds classified as either HA, MA or PA

This analysis showed that many of the compounds isolated from plant species in different plant orders were found to be PA. Exceptions to this were the plant orders Caryophyllales (n = 53), with 55% of its compounds found to be HA against *P. falciparum* D-S strains (Table 3). Similarly, against D-R strains, many compounds (45%) from the Caryophyllales (n = 62) were classified as HA. Likewise, most of compounds (46 to 75%) from the orders Chloranthales (n = 44), Buxales (n = 11) and Celastrales (n = 12) were classified as being HA against the D-R strains. Despite receiving considerable attention, the majority (ranging from 61 to 80%) of the compounds isolated from orders Asterales (n = 151), Gentianales (n = 192), Lamiales (n = 56), Sapindales (n = 168), Malpighiales (n = 86), Fabales (n = 138), and Magnoliales (n = 91) were classified as PA against D-S *P. falciparum* strains. This pattern was noted for the same orders, namely Asterales (n = 145), Gentianales (n = 190), Lamiales (n = 98), Sapindales (n = 287), Malpighiales (n = 186), Fabales (177), and Magnoliales (n = 117), against D-R strains with most of the compounds (ranging from 49 to 78%) being classified as PA. Generally, most of the compounds in all plant orders demonstrated an acceptable RI, i.e., ≤ 10. In addition to having most of their compounds classified as either HA and MA, remarkably, many molecules (> 70%) from the Buxales (n = 31), Chloranthales (n = 13), and Caryophyllales (n = 31) showed a good SI, i.e., ≥ 10 (Table 3).

Consistent with observations for the plant orders, most compounds isolated from most plant families were classified as PA (Table 4). In variance with this generalisation were the Ancistrocladaceae (n = 42) and Simaroubaceae (n = 19), from which most compounds (52 and 74%, respectively) were classified as HA against the D-S *P. falciparum* strains. The productivity of the Ancistrocladaceae (n = 45) and Simaroubaceae (n = 55) was retained against D-R strains with 49 and 62% of compounds from each respective plant family being classified as HA. Further plant families with most compounds (ranging from 45 to 75%) classified as HA against D-R were the Buxaceae (n = 11), Chloranthaceae (in Chloranthales) (n = 44), Celastraceae (in Celastrales) (n = 12), and Loganiaceae (in Gentianales) (41). Intriguingly, most compounds (63%) from the Loganiaceae (n = 84), were classified as PA against the D-S parasites. Despite receiving considerable research interest (as observed by the number of compounds isolated from them and screened for their antiplasmodial activity), many compounds isolated from the families Fabaceae (in Fabales), Rutaceae (in Sapindales), Rubiaceae (in Gentianales), Annonaceae (in Magnoliales) and Asteraceae (in Asterales) were classified as PA against both D-S and D-R *P. falciparum* parasites (Table 4).

**Table 4:**
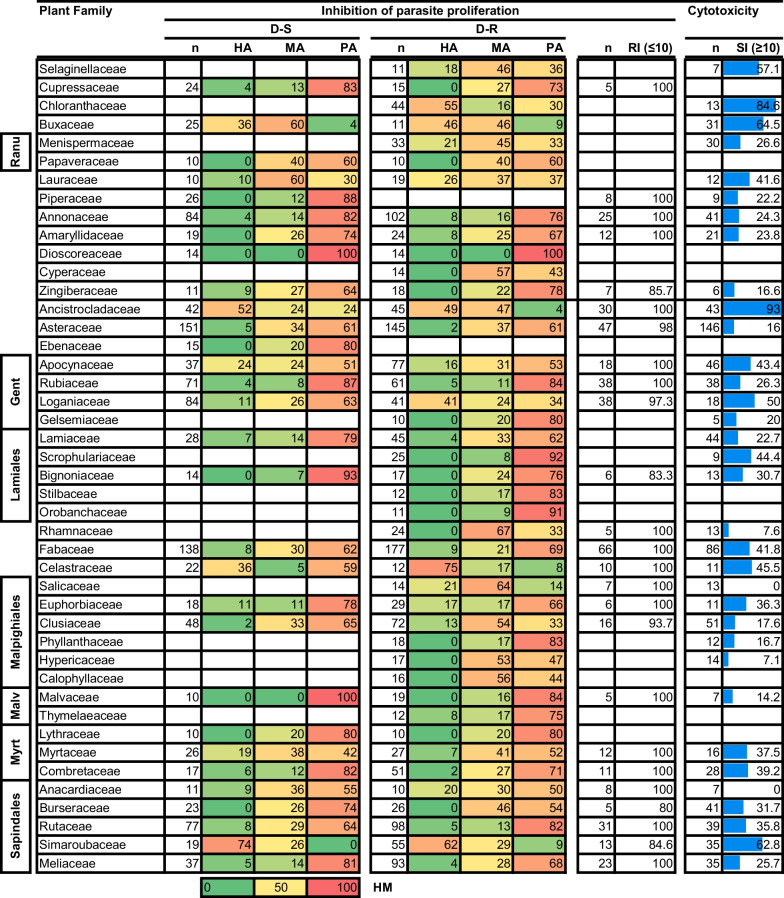
Activity and cytotoxicity of compounds isolated from plant species in respective plant families

n = number of compounds. HA, MA and PA values are expressed in % of total compounds evaluated. RI shows % of compounds with RI ≤ 10. SI shows % of compounds with SI ≥ 10. For RI and SI we have only presented data where ≥ 5 compounds have been evaluated and reported. *Myrt* Myrtales, *Malv* Myrtales, *Gent* Gentianales, *Ranu* Ranunculales. The heat map (HM) ranges from green (lowest value, 0%) to yellow (mid-range value, 50%) to red (highest value, 100%), visually illustrating the proportion of compounds classified as either HA, MA or PA

The resistance index for all plant families is good since > 90% of compounds have an RI < 10. Complementing the good activity profile of most compounds in the Ancistrocladaceae (n = 43), Buxaceae (n = 31), and Chloranthaceae (n = 17) was a good SI for most of them (> 60%) (Table 4).

From this expanded analysis, plants of the Buxales and Caryophyllales along with those in the families Simaroubaceae, Buxaceae, and Ancistrocladaceae have retained their ‘hot’ status. In addition to these, plants of the orders Chloranthales and Celastrales and the families Chloranthaceae and Celastraceae emerge as ‘hot’ taxonomic plant groups, particularly in producing potent compounds against the D-R *Plasmodium* parasites. It is quite striking to note that despite being most represented, the asterids and rosids (two major eudicot groups) only have one ‘hot’ plant order (the Celastrales) and two ‘hot’ families (the Simaroubaceae and Celastraceae) emerging from these lineages. It is noted though that generally, the HR for most compounds isolated from taxa in these clades is still relatively high, and noteworthy in relation to antimalaria drug discovery.

### Drug-likeness assessment of compounds produced by different higher plant orders and families

Potent antiplasmodial compounds should have good drug-like properties for ease of development into orally available preclinical and clinical candidates with reduced attrition rates in clinical trials. Drug-like properties include physicochemical descriptors including for example molecular weight (MW), consensus LogP (cLogP), hydrogen bond donors (HBD) and acceptors (HBA). Preference for drug discovery should be given to plant orders and families that produce compounds with good drug-like properties. To assess the drug-likeness of compounds isolated from the different plant orders and families, in silico calculated molecular and physicochemical descriptors of compounds were evaluated using different sets of criteria and by utilising data of clinically available antimalarial drugs (Tables 6 and 7). The analysis showed that a significant portion of these compound descriptors agreed with those of the criteria outlined by the Medicines for Malaria Venture, Lipinski’s Rule of 5 [27], Veber’s rules [28] and Ghose filters [29] indicating good characteristics of drug-likeness. However, some compounds isolated from the Buxales, Chloranthales, and Caryophyllales did not wholly fulfil the set criteria (Table 5). These discrepancies were noted for the respective families, which included the Buxaceae, Chloranthaceae and Ancistrocladaceae (Table 6). Out of the seven obtained physicochemical descriptors for compounds in these plant orders and families, some molecules did not fall within the specified set criteria for MW, the HBA, molar refractivity (MR), and cLogP.

**Table 5:** Calculated mean physicochemical descriptors for compounds isolated from different plant orders*

Plant Order	Physicochemical descriptors							PAINS	SA
	MW	RB	HBA	HBD	MR	TPSA	cLogP		
Lycopodiales	*547.4*	3.9	10.0	*5.1*	*147.5*	169.3	3.8	0.0	4.5
Cupressales	317.3	2.4	2.8	1.6	94.1	50.2	4.1	0.1	4.3
Ranunculales	426.2	2.0	5.7	0.5	124.0	56.2	3.7	0.1	4.6
Chloranthales	*599.9*	5.9	*10.2*	2.5	*151.2*	*154.1*	2.5	0.0	7.3
Buxales	*503.0*	5.3	4.0	2.0	*149.7*	60.2	*5.6*	0.0	6.4
Magnoliales	334.5	4.9	4.6	1.5	93.9	68.8	3.1	0.1	3.9
Laurales	345.7	4.1	4.7	0.9	98.8	55.0	3.1	0.0	4.2
Piperales	353.2	6.2	4.2	2.2	102.8	72.6	3.9	0.3	3.6
Dioscoreales	340.8	2.6	6.8	4.2	88.2	121.8	1.4	0.6	4.1
Asparagales	409.3	3.6	6.9	2.2	109.4	97.1	2.1	0.2	4.7
Zingiberales	307.6	3.9	3.5	1.2	87.2	55.2	3.2	0.0	5.1
Poales	271.3	2.1	3.6	1.8	77.2	57.7	2.9	0.0	3.7
Caryophyllales	497.5	4.2	7.0	3.2	*147.2*	95.8	4.2	0.3	5.0
*Asterids*									
Cornales	433.6	3.7	3.5	1.5	*130.2*	59.8	*5.2*	0.0	5.5
Ericales	*517.1*	4.3	6.6	3.6	*143.2*	109.4	4.3	0.1	5.7
Apiales	*524.7*	6.7	7.1	4.2	*143.3*	115.8	3.7	0.0	6.7
Asterales	392.9	5.6	6.2	2.1	104.9	96.5	2.8	0.1	5.1
Gentianales	393.0	2.9	4.4	1.7	115.5	67.6	2.8	0.3	4.8
Lamiales	374.0	4.6	6.3	3.1	98.8	101.9	2.0	0.3	4.8
*Rosids*									
Brassicales	430.0	6.4	7.1	3.6	118.6	112.4	2.7	0.1	4.9
Sapindales	410.2	4.4	6.1	1.8	111.2	91.4	2.9	0.1	5.1
Malvales	417.2	4.2	7.6	3.4	110.1	117.2	2.6	0.1	4.1
Myrtales	448.8	3.1	7.3	3.9	120.7	122.7	2.9	0.3	5.2
Fagales	*548.9*	7.8	*10.2*	4.9	*140.7*	*166.1*	2.3	0.4	6.1
Malpighiales	461.3	5.4	6.4	2.8	130.3	106.5	4.3	0.3	5.1
Rosales	*520.8*	6.1	6.0	2.6	*148.1*	102.6	4.8	0.1	5.7
Oxalidales	*519.8*	*11.4*	*12.2*	*7.1*	127.1	*205.8*	1.2	0.5	5.1
Fabales	361.1	4.1	5.3	1.8	101.0	77.9	3.2	0.1	4.2
Celastrales	*512.9*	8.3	4.6	2.6	*151.3*	79.2	*5.9*	0.0	6.4

*MW* molecular weight, *RB* rotatable bonds, *HBA* hydrogen bond acceptors, *HBD* hydrogen bond donors, *MR* molar refractivity, *TPSA* total polar surface area, *cLogP* consensus LogP, *PAINS* pan-assay interference compounds, *SA* synthesis accessibility

*Italic figures are those which don’t meet the set criteria

**Table 6:** Calculated mean physicochemical descriptors for compounds isolated from different plant families*

Plant Family	Physicochemical descriptors							PAINS	SA
	MW	RB	HBA	HBD	MR	TPSA	cLogP		
Selaginellaceae	*547.4*	3.9	10.0	5.1	*147.5*	*169.3*	3.8	0.0	4.5
Cupressaceae	317.3	2.4	2.8	1.6	94.1	50.2	4.1	0.1	4.3
Chloranthaceae	*599.9*	5.9	*10.2*	2.5	*151.2*	*154.1*	2.5	0.0	7.3
Buxaceae	*503.0*	5.3	4.0	2.0	*149.7*	60.2	*5.6*	0.0	6.4
*Ranu*									
Menispermaceae	461.0	2.2	6.1	0.6	136.0	58.1	4.0	0.0	5.1
Papaveraceae	343.5	1.2	5.4	0.5	95.7	55.5	2.8	0.1	3.3
Lauraceae	347.7	4.2	4.8	0.9	99.2	55.6	3.1	0.0	4.3
Piperaceae	354.2	7.1	4.0	2.3	104.5	70.8	4.3	0.3	3.6
Annonaceae	336.8	4.8	4.7	1.5	94.1	71.3	3.0	0.1	3.9
Amaryllidaceae	311.9	1.4	5.2	1.1	85.2	61.5	1.5	0.0	4.3
Dioscoreaceae	340.8	2.6	6.8	4.2	88.2	121.8	1.4	0.6	4.1
Cyperaceae	287.9	2.0	3.9	1.9	81.1	63.1	3.0	0.0	3.8
Zingiberaceae	307.6	3.9	3.5	1.2	87.2	55.2	3.2	0.0	5.1
Ancistrocladaceae	*507.4*	4.1	6.3	2.5	*154.9*	79.2	5.0	0.2	5.0
Asteraceae	392.9	5.6	6.2	2.1	104.9	96.5	2.8	0.1	5.1
Ebenaceae	378.6	3.2	2.7	1.3	113.4	47.1	*5.2*	0.1	4.8
*Gent*									
Apocynaceae	421.3	3.7	4.1	1.4	125.9	62.5	3.4	0.2	5.0
Rubiaceae	339.9	3.3	5.6	2.1	91.8	86.8	2.3	0.3	4.0
Loganiaceae	418.5	1.7	3.3	1.4	*130.3*	49.2	3.0	0.3	5.3
Gelsemiaceae	358.4	3.2	5.8	2.2	96.9	92.6	1.4	0.0	5.2
*Lamiales*									
Lamiaceae	302.6	2.7	4.2	1.8	84.0	68.0	2.6	0.5	4.2
Scrophulariaceae	494.0	8.0	*10.6*	*5.9*	119.9	169.2	0.1	0.1	6.2
Bignoniaceae	*509.0*	6.8	9.5	5.0	129.8	*156.9*	1.8	0.5	6.0
Stilbaceae	434.0	5.3	3.9	1.8	128.1	69.4	*5.4*	0.3	5.4
Orobanchaceae	365.1	4.3	7.7	4.5	90.5	127.1	0.7	0.2	4.8
Rhamnaceae	*550.1*	6.4	6.4	2.6	*155.8*	107.3	5.0	0.1	6.0
Fabaceae	361.1	4.1	5.3	1.8	101.0	77.9	3.2	0.1	4.2
Celastraceae	*512.9*	8.3	4.6	2.6	*151.3*	79.2	*5.9*	0.0	6.4
*Malpighiales*									
Salicaceae	*520.8*	*11.6*	7.5	0.8	*142.3*	100.8	4.7	0.0	6.9
Euphorbiaceae	471.2	5.6	8.8	2.9	122.5	*140.4*	2.4	0.3	5.1
Clusiaceae	465.8	5.4	6.3	3.4	134.3	110.4	4.8	0.4	5.0
Phyllanthaceae	*511.0*	7.7	6.5	3.3	*143.7*	112.6	4.3	0.1	5.7
Hypericaceae	383.7	3.9	5.2	2.7	110.9	88.3	3.9	0.3	3.7
Calophyllaceae	419.5	1.9	4.6	2.1	123.4	77.9	5.0	0.1	4.6
*Malv*									
Malvaceae	368.7	4.1	6.5	2.4	99.0	95.1	2.7	0.1	3.8
Thymelaeaceae	472.1	4.3	9.2	4.8	121.1	*149.6*	2.2	0.1	4.5
*Myrt*									
Lythraceae	*529.5*	1.6	*11.4*	*5.5*	*137.7*	*182.5*	2.0	0.4	5.6
Myrtaceae	455.1	3.3	6.8	2.8	123.7	112.8	3.5	0.3	5.7
Combretaceae	430.5	3.1	6.8	4.3	115.7	118.6	2.6	0.2	4.9
*Sapindales*									
Anacardiaceae	389.4	8.7	4.8	3.0	112.0	85.6	4.1	0.2	4.9
Burseraceae	337.7	2.0	2.8	1.2	101.1	45.5	4.4	0.1	5.0
Rutaceae	340.5	4.0	5.0	1.2	94.9	71.1	2.8	0.1	3.7
Simaroubaceae	459.6	2.9	9.3	3.6	110.9	*147.1*	0.4	0.0	6.2
Meliaceae	*512.8*	6.6	7.2	1.5	*138.8*	106.6	3.9	0.0	6.5

*MW* molecular weight, *RB* rotatable bonds, *HBA* hydrogen bond acceptors, *HBD* hydrogen bond donors, *MR* molar refractivity, *TPSA* total polar surface area, *cLogP* consensus LogP, *Myrt* Myrtales, *Malv* Myrtales, *Gent* Gentianales, *Ranu* Ranunculales, *PAINS* pan-assay interference compounds, *SA* synthesis accessibility

*Italics figures are those which do not meet the set criteria. *Myrt* Myrtales, *Malv* Myrtales, *Gent* Gentianales, *Ranu* Ranunculales

Further evaluation showed average descriptor values of many of the compounds from most plant families and orders compared well with those of approved antimalarial drugs. Similarly, not all antimalarial drug descriptors fell within the criteria for drug-likeness. For example, lumefantrine has a MW of 528.9, a MR of 152.6, cLogP of 7.9 and doxycycline and tetracycline both have 6 HBD and a TPSA of 181.6 A^2^.

Encouragingly, compounds from most of the plant orders and families were shown to be devoid of the pan-assay interference compounds (PAINS) substructures. The synthesis accessibility (SA) of many compounds from most plant orders and families was consistent with much of the currently available antimalarials. Notable exceptions were the compounds in the orders Buxales, Chloranthales, and Caryophyllales and the families Buxaceae, Chloranthaceae and Ancistrocladaceae. Their SA (ranging from 5 to 7.3) was in the same range as those of the artemisinin derivatives (6.5 to 6.7) (Tables 5 and 6). Most appealing will be plant orders and families which produce compounds which with a low SA value are easy to synthesise, exampled by the quinolines.

### Overall ranking to identify ‘hot’ higher plant orders and families for prioritisation in drug discovery projects

We formulated a rational ranking system which we used to identify the ‘hottest’ plant orders and families. We awarded points to different plant orders and families depending on how they performed in three attributes: the hit rate, HA for both D-S and D-R parasites, and the SI. We decided against including RI and drug-likeness in the ranking system as most of the compounds from most plant orders and families largely complied with set criteria for these indicators, so markedly reducing the value of these characteristics in improving selection resolution. Points were sequentially awarded based on the position of the taxonomic group in performance relative to other groups. For example, 1 point was given, per performance indicator, to the order or family with the highest hit rate, HA, and SI. The second, and third-positioned taxonomic groups were given 2 and 3 points, respectively. This scoring was repeated in that sequence until points were assigned to all plant families and orders. Thereafter, the total number of points was calculated for each family and order. We finally rationalised the number of points based on the number of indicators scored per taxonomic group resulting in the normalised points. This was done to ‘level’ the system, as not all taxonomic groups were scored for all indicators. We then ranked the taxonomic groups based on the number of points with the family or plant order, with the lowest number of normalised points ranking 1st and that with the greatest number of points ranking last (Tables 7 and 8 for orders and families respectively).

**Table 7:**
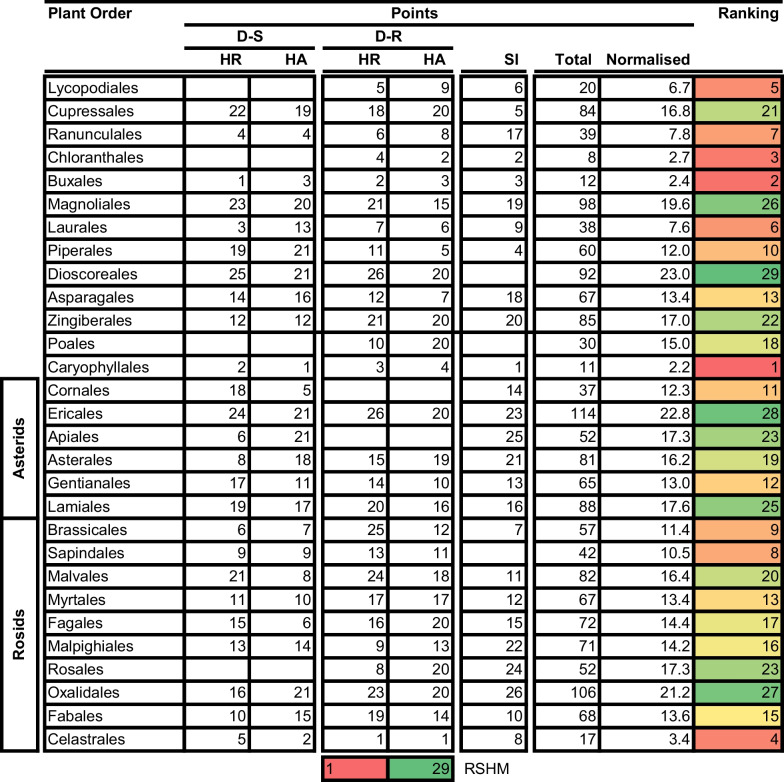
Ranking of plant orders for antimalarial drug discovery

*HR* Hit rate. Ranking score heat map (RSHM) ranges from red ‘hot’ (Best ranking, lowest points) to green (Lowest ranking, most points)

**Table 8:**
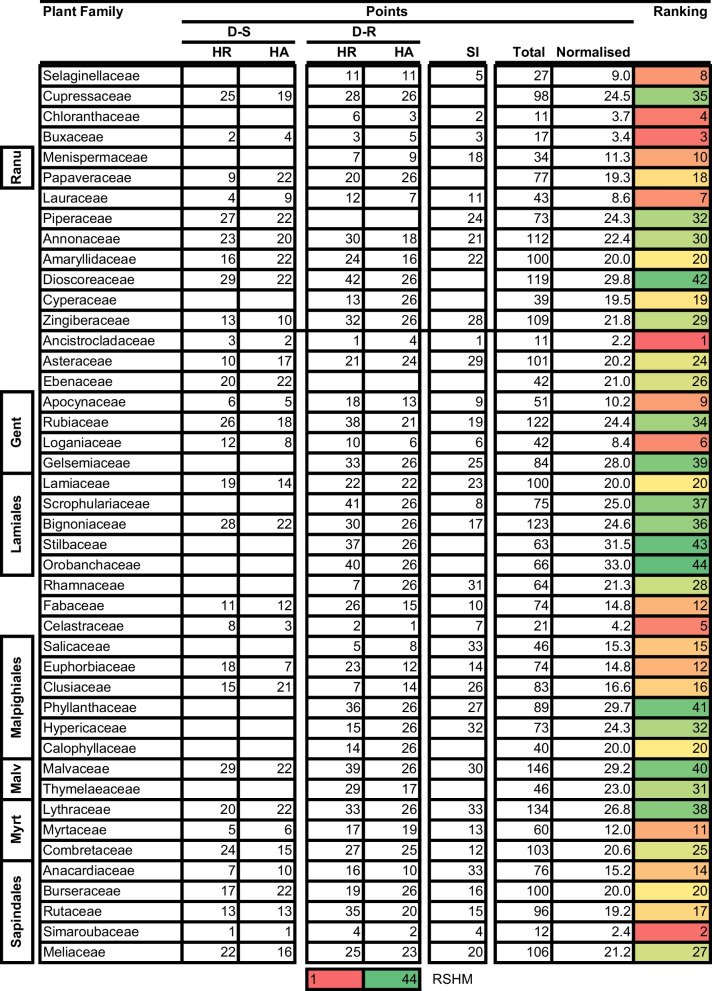
Ranking of plant families for antimalarial drug discovery

*HR* Hit rate. Ranking score heat map (RSHM) ranges from red ‘hot’ (Best ranking, lowest points) to green (Lowest ranking, most points). *Myrt* Myrtales, *Malv* Myrtales, *Gent* Gentianales, *Ranu* Ranunculales

Having adopted this ranking system, the following results were obtained and found to be consistent with earlier observations: the top 3 ranked orders were the (i) Caryophyllales, (ii) Buxales, and (iii) Chloranthales. The top 3 ranked plant families were the (i) Ancistrocladaceae (in Ancistrocladaceae), (ii) Simaroubaceae (in Sapindales), and (iii) Buxaceae (in Buxaceae) (Tables 7 and 8). The most prominent natural product classes found to be active (IC_50_ ≤ 10 µM) per each plant order and family were isoquinoline alkaloids and naphthoquinones (Caryophyllales and Ancistrocladaceae), steroid alkaloids and lupane triterpenoids (Buxales and Buxaceae), quassinoids (Simaroubaceae) and cycloeudesmane sesquiterpenoids (Chloranthales) (Table 9). We point out that majority of compounds classified by NPClassifier are manually classified as napthylisoquinoline (NIQs) alkaloids in their respective research publications, most of which emanate from the research group of Professor G. Bringmann [30]. Encouragingly, the HA compounds from these top plant taxa are structurally different to the ‘legacy’ antimalarial drugs [31].

**Table 9:** Natural compound classification of active compounds in ‘hot’ plant orders and families^#^

Natural product class (NPC)	Plant Order (% NPC composition)			Plant Family (% NPC composition)		
	Caryophyllales	Buxales	Chloranthales	Ancistrocladaceae	Simaroubaceae	Buxaceae
Unclassified*	17.1			21.9		
Unclassified^#^		4.2				4.2
Flavonols	2.4					
Isoquinoline alkaloids	43.9			40.6		
Naphthalenes and derivatives	2.4			37.5		
Naphthoquinones	31.7					
Tetraketide meroterpenoids	2.4					
Steroidal alkaloids		75.0				75.0
Lupane triterpenoids		12.5				12.5
Pregnane steroids		8.3				8.3
Cycloeudesmane^Ψ^			100.0			
Quassinoids					100.0	

^*,#^Compounds could only be assigned to Pathway and Super Class using the online tool NPClassifier [32] and not the natural product class. ^Ψ^Cycloeudesmane – cycloeudesmane sesquiterpenoids

The pressing need to discover and develop new antimalarials to mitigate drug resistance led us to consider the use of phylogenetic analysis coupled with bioactivity correlation to establish ‘hot’ plant orders and families worthy of prioritisation for antimalarial drug discovery projects. This endeavour has culminated in the identification of 3 ‘hot’ plant orders and families. One of the most intriguing findings of the current study was that the most promising plant orders and families are those from which either no antimalarial drug has previously been isolated or they are generally less studied (judging by either number of compounds isolated from them or number of publications reporting their investigation). In contrast, the families Asteraceae and Rubiaceae have received significant interest in their antiplasmodial evaluation. This interest we believe is driven by two factors, (i) previous discovery of antimalaria drugs from these families and (ii) their extensive use traditionally for malaria treatment as documented by ethnobotanical studies (e.g., [34, 35], and [36]). However, with a combined total of *ca.* 400 compounds isolated from the Asteraceae and Rubiaceae plant families, it is striking to note that only 16 of them (*ca*. 4%) have demonstrated IC_50_ values ≤ 1 µM either against D-S or D-R *Plasmodium* parasites. In contrast, of the less investigated plant families Simaroubaceae, Ancistrocladaceae, and Buxaceae, with a combined total of only 86 compounds isolated and screened for activity against the D-S *Plasmodium* strains, 45 of these compounds (53%) were reported to show IC_50_ values of ≤ 1 µM. Furthermore, the compounds isolated from these 3 families and those from other ‘hot’ taxonomic groups show good selectivity, outperforming the Asteraceae and Rubiaceae families (see Table 4).

From a medicinal chemistry perspective, emphasis is placed on the discovery of new compounds that occupy a chemical space that is different from that of the current clinically available antimalarial drugs. This chemical diversity brings with it a high likelihood of targeting novel biological space [37]. Having a unique target, compared to current antimalarials, increases the chances of the new scaffolds being potent against clinically drug-resistant *Plasmodium* strains. Our analysis shows that the HA compounds isolated from the ‘hot’ plant orders and families identified from the study occupy a different chemical space to current antimalarials providing further impetus to explore these taxonomic groups. For example, the most prolific compounds from the Simaroubaceae are classed as quassinoids, which the parasite has clinically not been exposed to [38]. Similarly, from the Buxaceae family, the most prolific compounds are steroidal alkaloids, which are chemically distinct from any of the clinically available curative malaria drugs [39].

The quassinoids class of natural products, which the Simarobuceae is well-established to produce, has also demonstrated in vivo antiplasmodial activity albeit with some level of toxicity noted. For example, the quassinoid bruceine B was shown to have an ED_90_ of 2.82 mg/kg/day. At a concentration threefold the ED_90_, bruceine B was observed to be 100% lethal against the mice used in the study [38]. Nonetheless, this compound was shown to be less toxic than other quassinoids, so raising hope that through medicinal chemistry less toxic but highly potent scaffolds from this natural product class could be synthesised. Notably, the synthesis of potent yet less in vivo toxic quassinoid analogues has been successfully undertaken for cancer studies [40]. NIQs have similarly shown exceptional in vivo activity. The NIQ dioncophylline C cured *P. bhergei*-infected mice following a single oral dose (50 mg/kg/day) with no observed toxicity [41]. While NIQs are structurally highly complex, approaches to their synthesis have been developed and comprehensively outlined [42] by the group of Professor G. Bringmann [30]. Moreover, simplified analogues of this class of compounds have been synthesised and proved to be potent against intra-erythrocytic asexual *Plasmodium* parasites [43]. Their clinical efficacy remains to be demonstrated.

It is interesting to note the generally high HR of most natural products isolated from many plant orders and families discussed in this study. These HR were substantially higher than those observed for synthetic compounds which have been described to be as low as 0.3% and 0.05% in some studies [44, 45]. However, caution needs to be exercised when considering these high hit rates for natural products. Firstly, the bioassay-guided assay approach is a popular approach used to isolate compounds from plants, where guidance is based on the observed bioactivity resulting in the isolation of bioactive molecules, albeit with varying potency. Secondly, the cut-off point (IC_50_ ≤ 10 µM) for the HR outlined in this paper is noted to be more tolerant than that used elsewhere, e.g., < 1.25 µM and ≤ 2 µM adopted by Plouffe et al*.* [45] and Dechering et al*.* [44], respectively. Nevertheless, the high HR is still encouraging, motivating for the continued investigation of plant-derived compounds to treat malaria.

In conclusion, given the need to accelerate antimalarial drug discovery, plants are a promising oasis deserving of continued investigation in this endeavor. Our study has shown that understudied plant orders and families are more deserving of intensified investigation in search of novel antimalarial drugs. We anticipate these findings will help direct researchers to focus and streamline their investigations on the few plant orders and families most likely to result in the discovery of highly active antiplasmodial compounds that can be channeled into medicinal chemistry programs.

## Material and methods

### Literature search

To identify published manuscripts for exploration in our study, we queried the PubMed database (https://pubmed.ncbi.nlm.nih.gov/) [46] searching for publications documenting antiplasmodial properties of compounds isolated from plants. The key phrase used was “*Plasmodium falciparum* and natural product” limiting the “Text Availability’ option to ‘Abstract”. The search was restricted to manuscripts published between 1964 and 2021. We then manually systematically screened the publications applying the following exclusion criteria.

i) Manuscripts documenting only antiplasmodial activity of compounds isolated from other natural sources, e.g., microorganisms, marine organisms, other than vascular (i.e., higher) plants were discarded.
ii) Manuscripts in which no compounds were isolated and screened were disregarded.
iii) Manuscripts in which only in vivo studies were carried out were excluded.
iv) Duplicate articles were excluded.

Articles subsequently remaining following the above process, were selected for the study.

### Taxonomic terminology

From the selected manuscripts, we manually collated compounds reported along with relevant pharmacological data, and species-of-origin saving this information on a Microsoft Excel spreadsheet. The captured data was verified 3 times to ensure all details collected were accurate and consistent with current nomenclature and taxonomy. Given disparities in taxon circumscriptions and related nomenclature inherent at all taxon ranks, we harmonised our systematic approach through aligning with the World Flora Online (WFO) database (http://www.worldfloraonline.org/) as of 7 November 2022, including in the assignment of species authors. In instances where the species authors for taxa had not been provided in the source pharmacological publications it was occasionally necessary, when more than one identical basionym exists, to resolve the identity of the research subject through consideration of the reported plant collection locality relative to data provided in the Global Biodiversity Information Facility (GBIF) (https://www.gbif.org/).

### Phylogenetic tree generation and data analysis

The phylogenetic trees were constructed as follows. Firstly, Text (.txt) files with the names of plant orders and families (as per WFO) were added onto the NCBI Taxonomic Browser ‘Common Tree’ [24]. The resulting tree was saved as a ‘Phyllip file’ (.phy) which was graphically displayed and manipulated using the iTOL online tool (v5) [25]. Here, default settings were used with only the following modifications made; Branch lengths—‘Ignore’ and Scaling factors—‘0.5 × horiz.’.

The SMILES of the compounds collated were either collected from databases including PubChem [47] and ChemSpider [48] or were generated from 2D structures drawn on ChemDraw Ultra (v8) [49]. Using the generated SMILES, compounds were classified into specific classes using the NPClassifier tool [32]. To evaluate drug-likeness we computed average physicochemical properties using the SwissADME online suite software [50]. Analysis, including calculation of mean values for RI, SI etc., was carried out using Microsoft Excel. Chemical space analysis was carried out on ChemPlot using structural similarity, PCA algorithm and scatter plot type options [33].

## Data Availability

The data that support the findings of this study are available from the corresponding author upon reasonable request.
